# Functional Analysis of the Promoter Region of Japanese Flounder (*Paralichthys olivaceus*) *β-actin* Gene: A Useful Tool for Gene Research in Marine Fish

**DOI:** 10.3390/ijms19051401

**Published:** 2018-05-08

**Authors:** Bo Wang, Huizhen Wang, Chen Gao, Yuxiang Liu, Chaofan Jin, Minmin Sun, Quanqi Zhang, Jie Qi

**Affiliations:** 1 Key Laboratory of Marine Genetics and Breeding, Ministry of Education, Ocean University of China, Qingdao 266003, China; wangbo@stu.ouc.edu.cn (B.W.); wanghuizhen_ouc@163.com (H.W.); 15063950079@163.com (C.G.); liuyuxiang@stu.ouc.edu.cn (Y.L.); 18663360681@163.com (C.J.); 17852726965@163.com (M.S.); qzhang@ouc.edu.cn (Q.Z.); 2 Laboratory for Marine Fisheries Science and Food Production Processes, Qingdao National Laboratory for Marine Science and Technology, Qingdao 266237, China

**Keywords:** *Paralichthys olivaceus*, β-actin promoter, transgenic stable cell, regulatory element, promoter activity

## Abstract

A newly isolated Japanese flounder (*Paralichthys olivaceus*) β-actin promoter and its derivative compact construct Poβ-actinΔ−1080/−801Δ−500/−201 have recently been demonstrated to promote ectopic gene expression in cell lines. Different Poβ-actin promoter deletion mutants were constructed and functionally characterized. Mutational analyses by dual-luciferase detected that three regulatory elements, including one enhancer (−1399/−1081) and two silencers (−1080/−801, −500/−201) in the first intron. The sequence located at −1399/−1081 was determined to significantly affect promoter activity. Additionally, the first exon (−1489/−1400) could also remarkably promote the β-actin promoter activity. In the following transduction application, we removed the two silencers and generated a compact reconstruct promoter/enhancer (Poβ-actinΔ−1080/−801Δ−500/−201), which exhibited relatively stronger promoter activity compared with Poβ-actin. Furthermore, the green fluorescent protein (GFP) transgenic stable flounder cell line was obtained by the reconstructed Poβ-actinΔ−1080/−801Δ−500/−201 promoter. Our study provided the potential application of Japanese flounder β-actin, particularly Poβ-actinΔ−1080/−801Δ−500/−201, in ectopic gene expression in the future.

## 1. Introduction

β-actin, the most abundant protein of eukaryotic cells, is extremely conserved during evolution [1]. Previous study showed that β-actin was engaged in key nuclear processes such as transcription, mRNA processing, and chromatin remodeling [2]. β-actin proteins participated in much more protein–protein interactions than other proteins, and showed important roles in many cellular functions, such as the maintenance of cell motility, cell shape, and polarity to the regulation of transcription [3]. Due to β-actin ubiquitous expression in all cell types, it was used as a reference gene in Northern blot analyses, Western blot analyses, and quantitative reverse transcription polymerase chain reaction (PCR) studies [4,5,6,7]. Additionally, β-actin promoters have been reported to be efficient ubiquitous regulatory elements and were widely used in transgenic mammalians [8], shrimp [9], and fish [10], and they were useful tools for transgenic studies in developmental biology.

Several experimental techniques, such as microinjection of embryos and gene knockdown/overexpression [11,12,13,14], have been successfully introduced into gene studies in different species, including tilapia [15], Atlantic salmon [16], zebrafish [17], rat [18], and mouse [19]. Additionally, the β-actin promoter from various fish species have been reported to be efficient ubiquitous regulators in the field of fish transgenesis. Studies of the zebrafish β-actin promoter showed that it could drive the *GFP* gene efficiently in zebrafish [20]. The β-actin promoter from medaka fish has also been isolated and used to drive a medaka *lacZ* gene and *GFP* gene in medaka embryos [21,22]. In addition, the carp β-actin promoter has been used successfully to drive reporter genes and protein coding sequences in various fish species [23,24]. Furthermore, previous evidence from transgenic animal studies have indicated that it is important and necessary for the development of transgenic species using gene regulatory materials originated from the same or closely-related species [23,25]. However, the studies on the expression system of marine fish is very limited. Most studies of certain genes in marine fish, such as Japanese flounder, tongue sole, and turbot, were focused on the genome-wide identification and evolutionary analysis [26,27,28]. Upon most occasions, the studies on gene functional research could only be carried out using other model species [29,30].

Promoter functions as the initiator of the gene expression, both in vitro and in vivo [31], and higher promoter activity allows for the use of plasmids at a decreased dose, potentially reducing the side effects of gene therapy [19]. The current research on the functions of certain genes in Japanese flounder has been hampered, because there have been no effective transgenic techniques in marine fish nor suitable promoters in terms of driving ectopic gene expression that have been established thus far. In this context, the aim of this study was to isolate a suitable and stable promoter for the gene function experiment of flounder by the transgene constructs which were “all flounder” in origin. We conducted the molecular cloning of 5′-flanking sequence of β-actin, which included 5′-upsteam sequence, 5′-untranslated exons, and the first intron. Furthermore, we investigated the regulatory regions of the Poβ-actin gene promoter, three essential cis regulatory regions were identified that might influence the activity of the Poβ-actin promoter. We found that the Poβ-actin promoter could drive the expression of exogenous genes in the flounder brain cell line (FBC) and the flounder embryo cell line (FEC). Our study provides a fundamental tool for the construction of gene function experiments in flounder cell lines, and it might be useful to study the targeted gene functions of flounder further.

## 2. Results

### 2.1. Isolation and Sequence Analysis of β-actin 5′-Flanking Region

**Figure 1:**
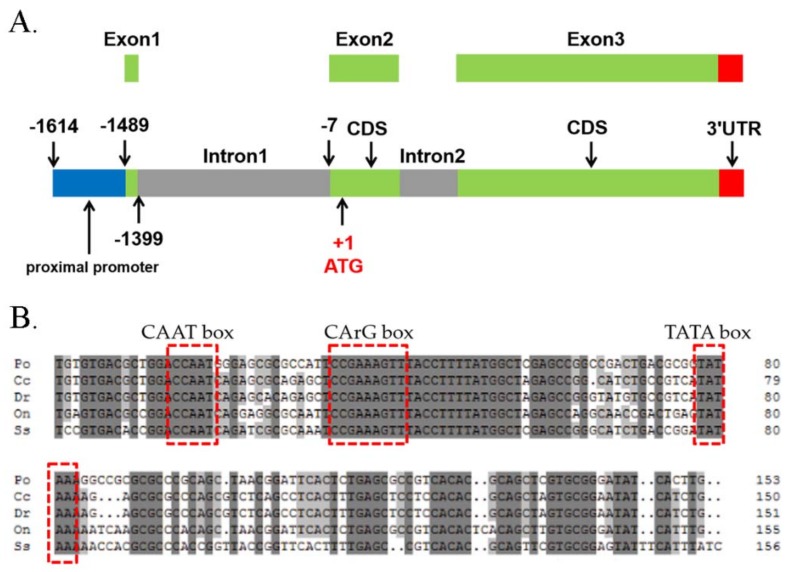
Nucleotide sequences of the fragment of Japanese flounder (*Paralichthys olivaceus*) *β-actin* gene. (**A**) A schematic map of the genomic sequence of flounder β-actin and the 5′-flanking sequences. The numbers indicate the positions (the first base of starting codon ATG was set as position +1). (**B**) Sequence alignment of the proximal promoter region is shown. Red and open boxes mark conserved sequences and known factor binding sites, respectively; CAAT-box, CArG motif, and TATA boxes.

**Figure 2:**
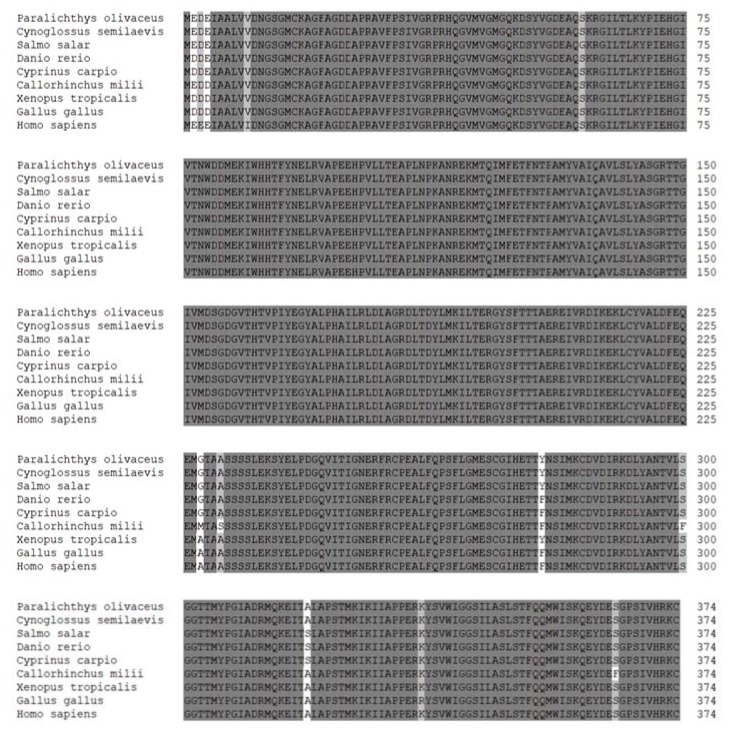
Multiple alignments of full-length β-actin amino acid sequences with eight other species. If 100% conservation exists in the seven sequences, then the amino acid shading is in black.

For promoter analysis, the translation initiation site (ATG) was designated as +1, and the upstream 5′-flanking sequence of Poβ-actin from −1614 to −1 bp was cloned and analyzed (GenBank accession number: MH036937). Alignment of the 5′-flanking sequence was carried out using the sequences from a previous study (GenBank accession number: HQ386788.1). The result showed that this newly-isolated 1.6 kb of sequences contains a proximal promoter, the untranslated exon1 and intron1, partial exon2 (Figure 1A), and sequences at the exon-intron boundary regions were well consistent with the GT-AG splicing rule [32]. Although the sequence of the promoter region and first intron were not as conserved as the coding region of Poβ-actin, which was highly conserved with other species by a BLASTn search (Figure 2), the canonical CAAT box, CArG motif, and TATA box were found in the promoter region of Poβ-actin and other selected fish species (Figure 1B and Figure S1). The accession numbers of sequences were given in Supplementary Materials (Table S2).

### 2.2. Analysis of the Proximal Promoter Regions

**Figure 3:**
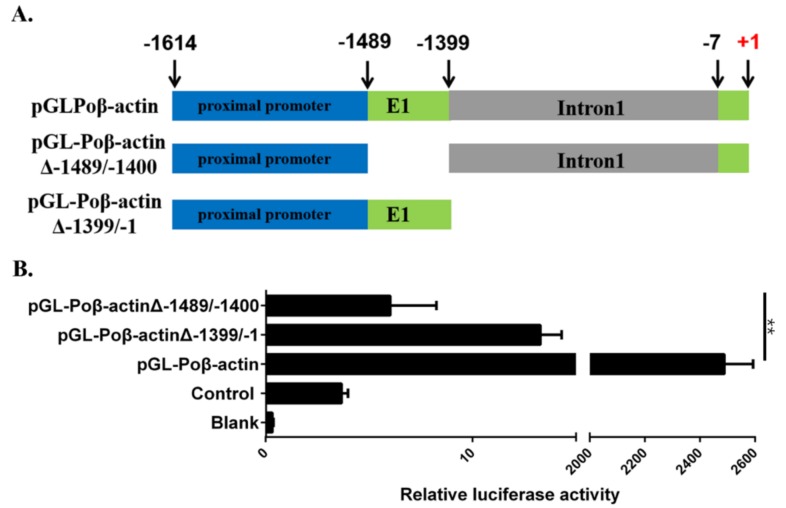
Examination of activity of 5′-upsream sequences, first exon and first intron of the Japanese flounder *β-actin* gene based on a reporter assay. (**A**) A schematic diagram of promoter region of luciferase reporter gene constructs. Showing various 5′-flanking sequence sequences of Japanese flounder *β-actin* gene fused with *luciferase* gene. (**B**) The relative levels of reporter gene expression in FEC cells are shown. The constructs were transiently cotransfected into cells along with pRL-tk control vector. The activity of firely and Renilla luciferase in the cell lysate were measured using a dual-luciferase reporter assay (Promega) at 48 h post transfection. Firefly luciferase activity was normalized to Renilla luciferase activity. The bars indicated mean ± S.D. of luciferase activity (*n* = 3). ** indicates statistical significance (*p* < 0.01).

To explore the promoter activity of newly isolated Poβ-actin sequence, several constructs containing various regions of the 5′-flanking sequence were generated and fused to a luciferase gene. An initial simple construct contained all 1614 bp of 5′-flanking sequences (construct named pGL-Poβ-actin, Figure 3A), which was compared with the luciferase reporter constructed with the deletion of the first extron (pGL-Poβ-actinΔ−1483/−1400) and the first intron (pGL-Poβ-actinΔ−1399/−1), respectively. The results showed that the proximal promoter was able to successfully drive the luciferase gene expression (Figure 3B). The first intron deletion (pGL-Poβ-actinΔ−1399/−1) results showed a severe reduction compared to that of the 5′-flanking sequence (pGL-Poβ-actin), and the deletion of the first exon promoter (pGL-Poβ-actinΔ−1483/−1400) which almost had no luciferase activity, indicating that the first intron and the first exon might play an important role in the Japanese flounder β-actin promoter region.

### 2.3. Functional Analysis of Japanese Flounder β-Actin Promoter Regulatory Regions

**Figure 4:**
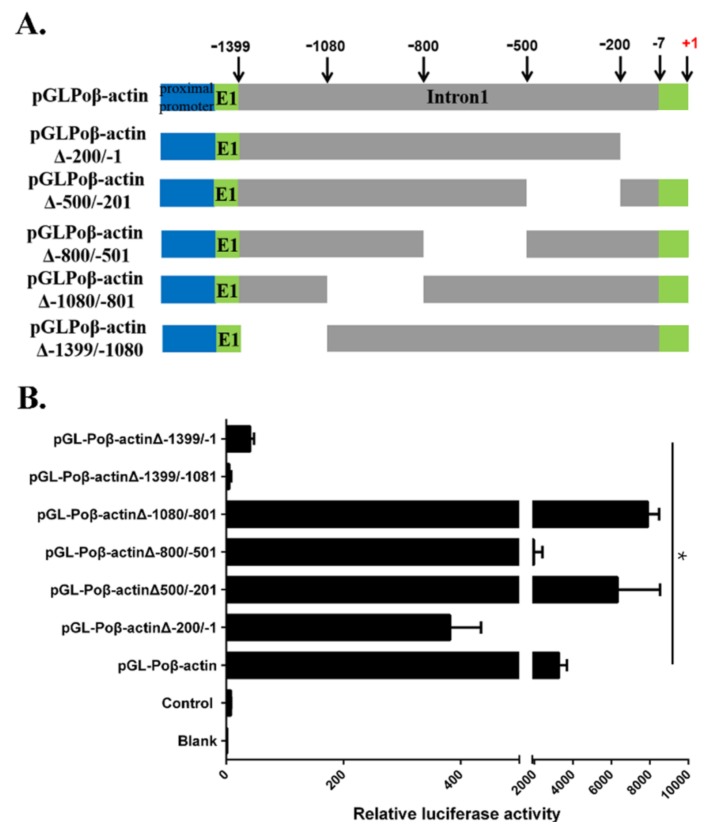
Characterization of regulatory region of 5′ -flanking sequences of the flounder *β-actin* gene based on the reporter assay. (**A**): schematic diagram of series deletion constructs with the luciferase reporter gene, which were made as described in the Materials and Methods. Δ indicates a deletion; and (**B**) the relative levels of reporter gene expression in FEC cells are shown. The constructs were transiently co-transfected into cells along with pRL-tk control vector. The activity of firely and Renilla luciferase in the cell lysate were measured using a dual-luciferase reporter assay (Promega) at 48 h post transfection. Firefly luciferase activity was normalized to Renilla luciferase activity. The bars indicated mean ± S.D. of luciferase activity (*n* = 3). * indicates statistical significance (*p* < 0.05).

To explore the regulatory element of Japanese flounder β-actin promoter in the first intron region, a series of deletion mutants within the 1.4-kb upstream sequence coupled to luciferase were constructed. The deletion mutants were based on pGL-Poβ-actin, and plasmids containing different 5′-flanking deletions of the Poβ-actin first intron region (Figure 4A). The activities of pGL-Poβ-actinΔ−1399/−1, pGL-Poβ-actinΔ−1399/−1081, pGL-Poβ-actinΔ−1080/−801, pGL-Poβ-actinΔ−800/−501, pGL-Poβ-actinΔ−500/−201, pGL-Poβ-actinΔ−200/−1 and pGL-Poβ-actin deletion constructs were then analyzed with their ability to drive the luciferase gene expression after they were transiently transfected into FBC cells (Figure 4B). The constructs with deletion of the sequence located between −1080/−801 (pGL-Poβ-actinΔ−1080/−801) and −500/−201(pGL-Poβ-actinΔ−500/−201) exhibited an increased luciferase activity compared to pGL-Poβ-actin, both of them showed an almost three-fold change of the luciferase activity compared to that of pGL-Poβ-actin. The pGL-Poβ-actinΔ−1080/−801 exhibited the highest luciferase activity among the deletion constructs. While other promoter mutants did not increase luciferase activity, the pGL-Poβ-actinΔ−1399/−1081 promoter mutant especially showed a significantly lower luciferase activity compared with the pGL-Poβ-actin construct promoter, and even lower than pGL-Poβ-actinΔ−1399/−1. Altogether, our results showed that there was more than one domain affecting the promoter activity of the putative Japanese flounder β-actin promoter (Figure 4B).

### 2.4. Expression of eGFP Using Japanese Flounder β-actin Promoter in FBC and FEC Cells

Two negative regulatory regions in the first intron region of Japanese flounder β-actin were identified according to serial deletion studies (Figure 4). For further understanding the Japanese flounder β-actin promoter on a transcriptional level, another reporter gene, *GFP*, was employed to examine the newly-isolated Japanese flounder β-actin promoters (eGFP-Poβ-actin) and its derived construct (eGFP-Poβ-actinΔ−1080/−801Δ−500/−201) with the negative regulatory regions deletion. The two Japanese flounder β-actin promoter fragments with 5′-end deletions were cloned into a pEGFP-1 vector, that is, without a promoter. The two constructed plasmids and the eGFP-C1 plasmid were transfected into FBC and FEC cells, respectively.

**Figure 5:**
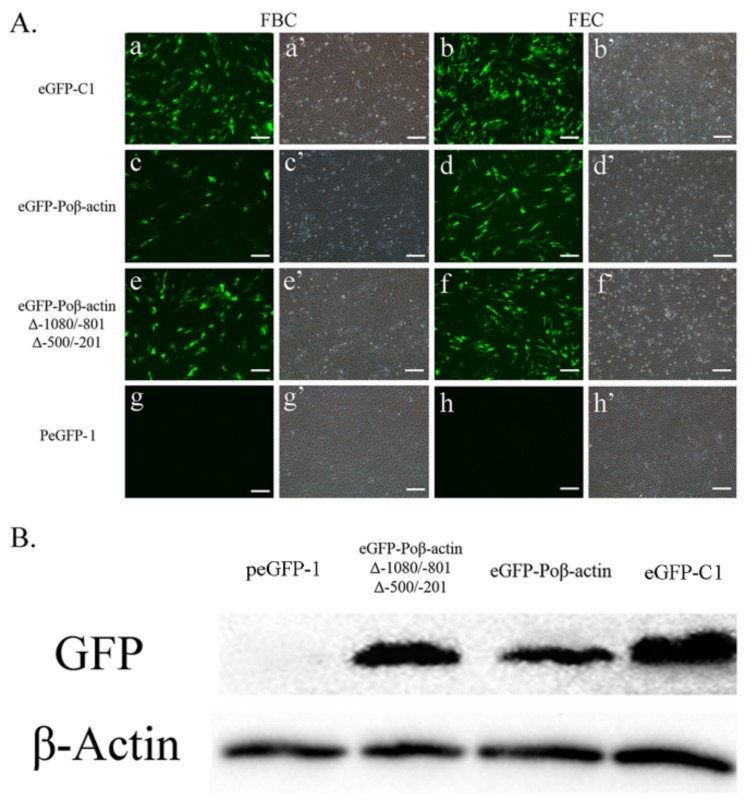
Expression of GFP driven by Poβ-actin Δ−1080/−801Δ−500/−201 in FBC and FEC cells. (**A**) The two type cells were transfected with eGFP-C1 (a, b); Poβ-actin-eGFP (c, d); Poβ-actinΔ−1080/−801Δ−500/−201-eGFP (e, f) and eGFP-1(g, h) were observed under a fluorescence microscope at day 2 post-transfection. The green fluorescence protein gene (EGFP) can be detected in a, b, c, d, e, and f (positive control), but not in g and h (negative control). The eGFP-C1 exhibited the highest expression of GFP in the both cell lines, and the GFP expression of eGFP-Poβ-actinΔ−1080/−801Δ−500/−201 was much higher than that of eGFP-Poβ-actin group. Bar = 100 μm. (**B**) The efficiency of different promoter was detected by Western blot in FEC cells, which were transfected with eGFP-1, eGFP-Poβ-actinΔ−1080/−801Δ−500/−201, eGFP-Poβ-actin and eGFP-C1, respectively.

The GFP expression was assayed 48 h after transfection and the eGFP-positive cells of each sample were counted in three images under identical conditions using an inverted fluorescence microscope. Among these three groups, the eGFP-C1 plasmid exhibited the highest expression of eGFP in both FBC and FEC cells (Figure 5A), and the GFP expression of eGFP-Poβ-actinΔ−1080/−801Δ−500/−201 was much higher than that of eGFP-Poβ-actin group. The efficiency of the three kinds of promoter in FEC cells are also shown by Western blot (Figure 5B), which was consistent with green florescence detection results.

### 2.5. Screening of eGFP Overexpression in Stable Cell Line

**Figure 6:**
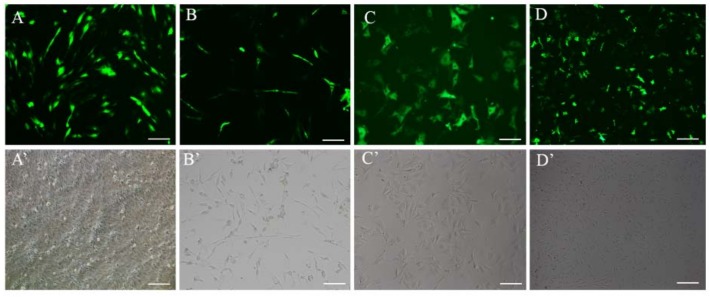
Long-term stability and expression experiments. (**A**) The FEC cells were transfected Poβ-actinΔ−1080/−801Δ−500/−201-eGFP on day 2; (**B**) two weeks after the G418 antibiotic was used in the transfected FEC cells and the proportion of GFP overexpression cells is significantly increased; (**C**,**D**) The best cell colony with GFP florescence following expending culture and almost all cells had high fluorescence emission. The GFP overexpression stable FEC cell line cultured until week 4 and month 2 in medium without G418, respectively. Bar = 100 μm.

To screen the eGFP overexpression in a stable cell line, we transfected the eGFP-Poβ-actin Δ−1080/−801Δ−500/−201 vector into FEC cells. Following transfection, 600 μg/mL of G418 were used to screen the eGFP overexpression the FEC cell line, which was the most optimal concentration of G418 for treated FEC cells, as was shown in Figure S2. The DMEM/F12 medium was replaced with fresh medium containing 600 μg/mL G418 every three days. The G418 antibiotic was used for two weeks (Figure 6B), and fluorescence was monitored every time after the DMEM/F12 medium was changed. Then the surviving cells were cultured with DMEM (10% FBS) containing 300 μg/mL G418. The DMEM medium was changed every three days until the cells had grown into a colony. After two weeks, cell fluorescence was observed using microscopy, and the best colony in which almost all cells had high fluorescence emission was labeled and expanded gradually using a 24-well plate (Figure 6C). Additionally, as shown in Figure 6D, a stable expression cell line of eGFP was provided for at least two months.

## 3. Discussion

The vectors that could express high levels of heterologous genes are important for transgenic applications, and many factors significantly affect a successful expression vector. Previous study has shown that endogenous promoters could drive higher levels of expression [33]. Although the Japanese flounder *β-actin* gene has been reported as a reference gene in the literature [34,35], little was known about the functional characterization of its promoter region. In this study, a series of experimental tests were performed to gain more insight into the application of the Poβ-actin promoter and its compact construct in foreign gene expression research. We cloned 5′ flanking region of Poβ-actin ranging from −1614 to −1 bp in Japanese flounder to investigate the function and regulation of Poβ-actin promoter. Then we evaluated the reconstructed Poβ-actin promoter strength by testing its function along with CMV promoter in two flounder cell lines. Based on the dual-luciferase activity, we proved that Poβ-actin promoter has been active in driving luciferase expression in transfected FBC cells. In addition, since the tilapia β-actin promoter has been successfully employed in fish transgenic research [36], our findings suggested that the reconstructed Poβ-actin promoter has the potential to be applied in fish transgenic research.

The sequence located at −1614 to −1461, including CAAT, CArG motif, and TATA Box, were identified as essential domains for promoter activity. Previous study has shown that the serum-response element CArG motif was positioned in the carp β-actin promoter region between CAAT and TATA box [36,37]. In the present study, the flounder β-actin proximal promoter contains the typical CAAT box at position −1585, a TATA box at position −1522, and a CArG motif at position −1566, which showed a similar relative position with tilapia and carp [36,37].

**Figure 7:**
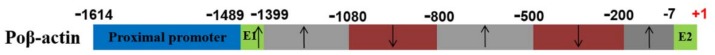
Summary of the Japanese flounder *β-actin* gene regulatory regions. The regulatory loci identified in this report are designated by ↑ when the gene expression is being enhanced, and ↓ when gene expression is being silenced. The numbers indicate the positions (the first base of starting codon ATG was set as position +1).

To further characterize the regulatory element of flounder β-actin promoter, we explored the contributions of the six regions of the promoter with respect to their abilities to modulate gene expression according to present study (Figure 7). Previous studies indicated that the first intron enhancer elements were required for the strong activity of the β-actin promoter in other species [38,39]. Our result also demonstrated that the first intron is necessary for activity of the β-actin promoter, as the whole first intron deletion mutant Poβ-actinΔ−1399/−1 showed much lower promoter activity (Figure 4). Further, different Poβ-actin promoter deletion mutants of the first intron were constructed and functionally characterized. The promoter deletion mutants Poβ-actinΔ−1080/−801 and Poβ-actinΔ−500/−201 showed much higher promoter activity than Poβ-actin in tested cell lines (Figure 4), and other deletion mutants showed lower promoter activity, especially Poβ-actinΔ−1399/−1081. This dual-luciferase analyses results suggested that three regulatory elements, including one enhancer (−1399/−1081) and two silencers (−1080/−801, −500/−201), were also detected in the first intron, which was consistent with a study on shrimp β-actin [9]. Additionally, the sequence located at −1399/−1081 was determined to affect promoter activity most significantly. Moreover, the first exon region (−1489/−1400), has shown to significantly promote the β-actin promoter activity (Figure 3). This observation, to the best of our knowledge, has not yet been previously reported.

Many species’ β-actin promoters have been used in transgenic research, which are known to have strong promoter activity in mammal, fish, and shrimp [9,40,41]. Then we evaluated Poβ-actin promoter strength by testing its function, along with CMV promoter, in two flounder cell lines. The reconstruct promoter/enhancer (Poβ-actinΔ−1080/−801Δ−500/−201) exhibited a much higher activity than that of Poβ-actin promoter in FBC cells, as shown by Western blot, and a little lower activity than CMV promoter. Moreover, we successfully obtained the GFP overexpression-stable FEC cell line by the reconstructed Poβ-actin promoter/ enhancer using G418 [42], which makes it available for the construction of gene function experiments in flounder cell lines. To the best of our knowledge, this is the first report about transgenic stable cell lines obtained in marine fish. The result suggested the β-actin promoter/enhancer (Poβ-actinΔ−1080/−801Δ−500/−201) has the potential as an efficient and stable tool for transgenic application in marine fish. Altogether, present studies have clearly demonstrated that the β-actin promoter/enhancer is more acceptable to consumers, and pointing out the reconstructed Poβ-actin promoter/enhancer would be an ideal promotion for Japanese flounder transgenic research in the future.

In conclusion, our study demonstrated the characteristics of both Poβ-actin and Poβ-actin promoter/enhancer, and confirmed the possibility of using Poβ-actin promoter/ enhancer for foreign gene expression in cell lines. It is an ideal promoter for the construction of an expression vector in the application of gene transfer in Japanese flounder and other teleosts. Additionally, our results provide a basis for further studies in understanding Poβ-actin regulatory mechanisms in Japanese flounder.

## 4. Materials and Methods

### 4.1. Experimental Fish

Japanese flounder healthy adults were obtained from a commercial hatchery located in Haiyang City of China and cultured for one week in seawater tanks at 20 ± 1 °C under laboratory conditions. Animal experiments were all conducted in accordance with the Regulation for the Administration of Affairs Concerning Experimental Animals (China, 1988). The research was also approved by College of Marine Life, Ocean University of China (Qingdao, China).

### 4.2. Cell Line Culture

The continuous FBC and FEC were obtained from brain and embryos of Japanese flounder and maintained using the method according to Tong et al. [43]. The monolayer cultures of the cells exhibited an epithelioid morphology and they have been subcultured for >200 passages. All cells were maintained in Dulbecco’s modified Eagle’s medium, DMEM/F12 (1:1) (Gibco, Carlsbad, CA, USA) and supplemented with 10% fetal bovine serum (FBS; Gibco; Thermo Fisher Scientific Inc., Waltham, MA, USA) at 24 °C in an atmosphere of air [44]. The cell culture medium contained 100 μ/mL penicillin and 100 μg/mL streptomycin.

### 4.3. Cloning of Poβ-actin Promoter Sequence

Genomic DNA was extracted from the muscle tissue of Japanese flounder using the traditional phenol/chloroform method [45]. The promoter region of Poβ-actin and the *Po-β-actin* gene were synthesized from the genomic DNA and cDNA of Japanese flounder through polymerase chain reaction (PCR) (Novoprotein, Shanghai, China), respectively. A specific primer pair consisting of a forward primer with Kpn I site and a reverse primer with Hind III site (Table S1) was designed to amplify a 1.6 kb-long DNA fragment (−1614 to −1 bp, +1 corresponding to the start codon “ATG”). Both of the two sequences were performed in a mixture with a total volume of 25 μL. After an initial 5 min denaturing step at 95 °C, 35 cycles of amplification were performed for 30 s at 95 °C, 30 s at 58 °C, and 2 min at 72 °C, successively, and then followed by a final extension for 10 min at 72 °C. The amplified PCR products were subcloned into a pMD19-T vector (Takara, Dalian, China) and sequenced.

### 4.4. Sequence Analysis

The potential transcription factor binding sites in the 5′-flanking region of *β-actin* gene of Japanese flounder and other vertebrates were predicted by using the online program MatInspector (http://www.genomatix.de/). The conserved DNA motifs in the 5′-flanking regions Japanese flounder were identified by online program Dialign from the Genomatix suite (http://www.genomatix.de/).

### 4.5. Construction of Promoter-Luciferase Cassette

The pGL3-Poβ-actin were generated from the −1614/−1 of Poβ-actin promoter sequence using specific primers (Table S1). The primer pairs with restriction sites (Kpn I and Hind III) were used to generate the reporter gene constructs. The amplified PCR products were examined through 1.5% agarose gel electrophoresis and purified using the Zymoclean Gel DNA Recovery Kit (Zymo Research, Orange, CA, USA). And the target PCR product were digested and then inserted into pGL3-basic luciferase vector (Promega, Madison, WI, USA). A series of deletion plasmids were constructed, including pGL-Poβ-actinΔ−1399/−1, pGL-Poβ-actinΔ−1399/−1081, pGL-Poβ-actinΔ−1080/−801, pGL-Poβ-actin Δ−800/−501, pGL-Poβ-actinΔ−500/−201, pGL-Poβ-actinΔ−200/−1, pGL-Po β-actinΔ−1489/−1400, and pGL-Poβ-actin. Those plasmids were generated from the pGL3-Poβ-actin via PCR according to the protocol of the QuikChange site-directed mutagenesis kit (Agilent Technologies, Santa Clara, CA, USA). The specific mutagenic primers (Table S1) were designed according to the cloned sequence with Integrated DNA Technologies (IDT) website (http://sg.idtdna.com/Primerquest/ Home/ Index). The PCR program was as follows: 95 °C for 5 min and 30 cycles of 95 °C for 30 s, 58 °C for 30 s, and 72 °C for 7 min. The PCR products were subjected to DpnI (NEB, Beijing, China) digestion to eliminate the parent vector. All the mutant plasmids were sequenced to verify that no other mutation occurred during PCR amplification.

### 4.6. Construction of EGFP Plasmid

To explore the relative strength of promoter activity, the sequences of Poβ-actin, were generated from the −1614/−1 construct via PCR using specific primers (Table S1). Different promoter deletion construct Poβ-actinΔ−1080/−801Δ−500/−201 was generated from the Poβ-actin construct via PCR using two specific primers (Table S1).

### 4.7. Transient Transfection

A day before transfection, the cells were plated in 24-well tissue culture plates at 2 × 10^5^ cells/well. The plated cells were then cultured in 0.5 mL of DMEM medium at 24 °C. Transient cell transfection was performed using Lipofectamine 3000 (Invitrogen, Carlsbad, CA, USA) according to the manufacturer’s protocol when the cells were grown to 80–90% confluence.

For dual-luciferase reporter assay, each well was cotransfected with 500 ng of Poβ-actin promoter/pGL3-basic constructs (deletion mutation constructs), 10 ng of pRL-TK plasmid (Promega, Madison, WI, USA), which served as an internal control to ensure the transfection efficiency, 1 μL of P3000 and 1.5 μL of Lipo3000. The control cells were transfected with the pGL3-basic plasmid, which was a promoterless vector. The medium of each well was replaced with 500 μL of growth media containing 10% FBS after 6 h of transfection. The transfection was maintained for 24 h. The cells were washed with phosphate-buffered saline (PBS) and lysed with 100 μL of Passive Lysis Buffer (plb) provided in a dual-luciferase reporter assay system (Promega, Madison, WI, USA) for 15 min. Luciferase activity was measured with GloMax 20/20 Luminometer (Promega, Madison, WI, USA). The luminometer was programmed to perform a 2-s pre-measurement delay, followed by a 10-s measurement period for each reporter assay. Twenty (20) microliters of cell lysate was transferred into the EP tube containing 100 μL of Luciferase Assay Reagent II (LAR II) and firefly luciferase activities were measured. Then, 100 μL of Stop and Glo^®^Reagent (Promega, Madison, WI, USA) was added and the Renilla luciferase activities were read. The luciferase activities were obtained by the ratio of firefly to Renilla luciferase. All of the data were measured from three independent experiments.

For the GFP reporter assay, each well was transfected with 500 μL of transfection mixture containing 500 ng of Poβ-actin promoter/pEGFP-1 plasmid (Clontech, Dalian, China). The positive control cells were transfected with the vector driven by the CMV promoter (pEGFP-C1) to determine transfection efficiency. In addition, the negative control cells were transfected with the promoterless vector pEGFP-1 (Clontech, Dalian, China). The transfection was maintained for 48 h. The GFP-positive cells of each sample from three independent experiments were counted under an inverted fluorescence microscope.

### 4.8. Screening of Poβ-actin-EGFP Overexpression Cells

To determine the optimal concentration of Geneticin (G418) (Hanheng Biotechnology, Shanghai, China) for FEC cells, the cells were passaged in 24-well plate. G418 selection was started at two days after cell passage. Different dosages of G418 arranging from 0 to 900 μg/mL were used to incubate with the cells. Then the proportion of cell death was counted under an inverted microscope (Ti-E/Ti-U; Nikon, Tokyo, Japan). After 15 days, the lowest concentration that caused FEC cells all death was selected as the optimal concentration of G418 for treated FEC cells.

Then the eGFP-Poβ-actinΔ−1080/−801Δ−500/−201 plasmid was transfected into the FEC cells using Lipofectamine^®^3000 (Invitrogen; Carlsbad, CA, USA). The cells were incubated at 24 °C in complete DMEM/F12 medium containing optimal concentration G418 for 14 days for selection of positive cells. The Cells were then cultured at 24 °C in DMEM/F12 medium containing half of optimal concentration G418. The transfection efficiency and purity of positive cells were evaluated according to the presence of green florescence, and the positive clones were selected. Cells were cultivated for two months without selection medium and the stability of the transfected vectors were monitored.

### 4.9. Statistical Analysis

The data of the luciferase assays were tested using Excel and SPSS (19.0, IBM, New York, USA). The significance of the differences among the samples were calculated using one-way analysis of variance (ANOVA) followed by Duncan’s test. All data were expressed as mean ± standard deviation. *p* values of < 0.05 were considered statistically significant.

## Supplementary Materials

Supplementary materials can be found at http://www.mdpi.com/1422-0067/19/5/1401/s1.

## Abbreviations

CMV	CytoMegalo Virus
GFP	Green Fluorescent Protein
IDT	Integrated DNA Technologies
PCR	Polymerase Chain Reaction
FEC	Flounder Embryo Cell line
FBC	Flounder Brain Cell line
DMED/F12	Dulbecco’s Modified Eagle Media: Nutrient Mixture F-12
FBS	Fetal Bovine Serum
PBS	Phosphate Buffered Saline
