# Supplementary material for: Functional Analysis of the Promoter Region of Japanese Flounder (Paralichthys olivaceus) β-actin Gene: A Useful Tool for Gene Research in Marine Fish
Source: Int J Mol Sci. 2018 May 8;19(5):1401. doi: 10.3390/ijms19051401 (PMC5983668; doi:10.3390/ijms19051401)
Supplement: Supplementary file 1 [file ijms-19-01401-s001.pdf]

International Journal of *Molecular Sciences*

MDPI

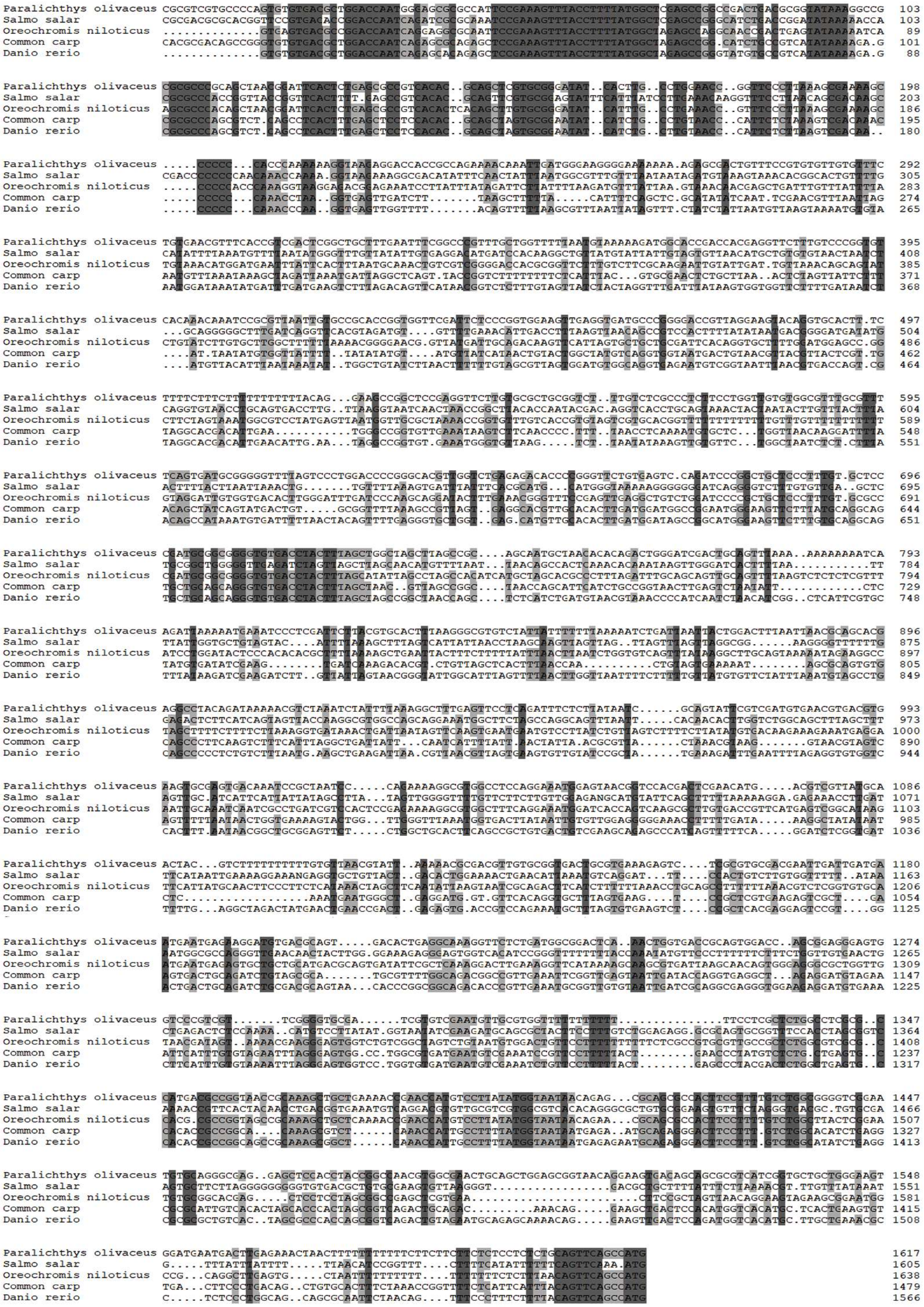

**Figure S1.** Multiple alignment of β-actin promoter. Numbering of the nucleotide sequences is given on the left.

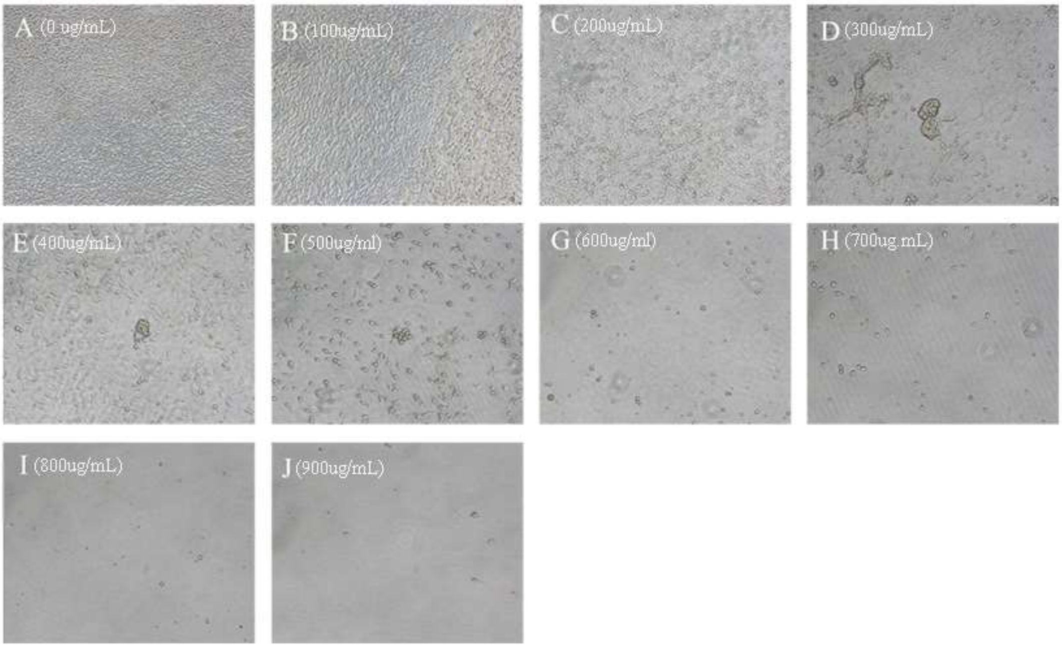


**Figure S2.** The screening of optimal concentrations of G418 for FEC cells. The FEC cells were passaged in a 24-well plate with different concentrations of G418 (arranged from 0 to 900 μg/mL) for two weeks.

**Table S1.** List of primer pairs used in the amplification of promoting mutation.

| Primer name | Sequence |
|---|---|
| Poβ-actin-Fw(KpnⅠ) | CGGGGTACCCGCGTCGTGCCCCAGTGT |
| Poβ-actin-Rv(HindIII) | CCCAAGCTTGGCTGAACTGCAGAGAGGAGAGA |
| Poβ-actin-EGFP-Fw(HindIII) | CCCAAGCTTCGCGTCGTGCCCCAGTGT |
| Poβ-actin-EGFP-Rv(KpnⅠ) | CGGGGTACCGGCTGAACTGCAGAGAGGAGAGA |
| Del-1~-200-Fw | ggtaataacagagcgcaagcttggcattccgg |
| Del-1~-200-Rv | ccggaatgccaagcttgcgctctgttattacc |
| Del-201~-500-Fw | gcaactacgtctttttttttttgtgttaacgtagcgccacttcctttt |
| Del-201~-500-Rv | aaaaggaagtggcgctacgttaacacaaaaaaaaaaagacgtagttgc |
| Del-501~-800-Fw | caagattaaaaatgaaatccctcattaaaaacgcgacgttgtgcgg |
| Del-501~-800-Rv | ccgcacaacgtcgcgtttttaatgagggatttcatttttaatcttg |
| Del-801~-1080-Fw | aggaagccggctccggattcttacgtgcac |
| Del-801~-1080-Rv | gtgcacgtaagaatccggagccggcttcct |
| Del-1081~-1399-Fw | cccccacccaaaaaagaggttcttgtgcgctg |
| Del-1081~-1399-Rv | cagcgcacaagaacctcttttttgggtggggg |
| Del-1400~-1614-Fw | tctatcgataggtaccgtaagaggaccaccgc |
| Del-1400~-1614-Rv | gcggtggtcctcttacggtacctatcgataga |
| Del-1~-1399-Fw | ccccccacccaaaaaagaagcttggcattccg |
| Del-1~-1399-Rv | cggaatgccaagcttctttttttgggtgggggg |
| Del-1400~-1483-Fw | taacggattcactctggtaagaggaccaccgc |
| Del-1400~-1483-Rv | gcggtggtcctcttaccagagtgaatccgtta |

**Table S2.** The sequences used for multiple alignments.

| Name | GenBank accession number and Ensembl number |
|---|---|
| Common carp β-actin gene promoter region | M24113.1 |
| Danio rerio β-actin gene promoter region | EF026002.1 |
| Oreochromis niloticus β-actin gene promoter region | AY116536.1 |
| Paralichthys olivaceus β-actin gene promoter region | MH036937 |
| Salmo salar β-actin gene promoter region | DQ924958.1 |
| Paralichthys olivaceus β-actin gene | HQ386788.1 |
| Paralichthys olivaceus β-actin protein | ADP88939.1 |
| Cynoglossus semilaevis β-actin protein | NP_001295108.1 |
| Salmo salar β-actin protein | ACI67042.1 |
| Danio rerio β-actin protein | NP_853632.3 |
| Cyprinus carpio β-actin protein | AAA68886.1 |
| Xenopus tropicalis β-actin protein | NP_998884.1 |
| Gallus gallus β-actin protein | CAA25004.1 |
| Homo sapiens β-actin protein | AAH15695.1 |
| Callorhinchus milii β-actin protein | NP_001279672.1 |
